# Immunization Coverage in Migrant School Children Along the Thailand-Myanmar Border

**DOI:** 10.1007/s10903-015-0294-x

**Published:** 2016-10-01

**Authors:** Aiko Kaji, Daniel M. Parker, Cindy S. Chu, Wipa Thayatkawin, Jiraporn Suelaor, Rachai Charatrueangrongkun, Kloloi Salathibuppha, Francois H. Nosten, Rose McGready

**Affiliations:** 1 Department of Global Community Health and Behavioral Sciences, School of Public Health and Tropical Medicine, Tulane University, New Orleans, LA USA; 2 Shoklo Malaria Research Unit, Mahidol-Oxford Tropical Medicine Research Unit, Faculty of Tropical Medicine, Mahidol University, Mae Sot, Tak Thailand; 3 Mahidol-Oxford Tropical Medicine Research Unit, Faculty of Tropical Medicine, Mahidol University, Bangkok, Thailand; 4 Centre for Tropical Medicine, Nuffield Department of Medicine, University of Oxford, Oxford, UK

**Keywords:** Immunization, Vaccine coverage, Migrant children, School

## Abstract

The objective of this project was to document and increase vaccine coverage in migrant school children on the Thailand-Myanmar border. Migrant school children (n = 12,277) were enrolled in a school-based immunization program in four Thai border districts. The children were evaluated for vaccination completion and timing, for six different vaccines: Bacille Calmette-Guerin (BCG); Oral Polio vaccine (OPV); Hepatitis B vaccine (HepB); Diphtheria, Pertussis and Tetanus vaccine (DTP); Measles Containing Vaccine or Measles, Mumps and Rubella vaccine (MMR); Tetanus and Diphtheria containing vaccine (Td). Vaccine coverage proportions for BCG, OPV3, DTP3, HepB3 and measles containing vaccine were 92.3, 85.3, 63.8, 72.2, and 90.9 % respectively. Most children were able to receive vaccines in a time appropriate manner. School-based immunization programs offer a suitable vaccine delivery mechanism for hard-to-reach populations. However, these data suggest overall low vaccine coverage in migrant populations. Further efforts toward improving appropriate vaccine coverage and methods of retaining documentation of vaccination in mobile migrant populations are necessary for improved health.

## Introduction

Immunization is one of the most successful and cost-effective health interventions of modern medicine, preventing an estimated 2.5 million deaths each year [1]. Vaccine preventable diseases and disabilities have decreased drastically over the last several decades, resulting in healthier children and, subsequently, adults [2]. Despite this progress, vaccine preventable diseases are still a major cause of morbidity and mortality in low and middle income countries [1] and several studies [3–5] have investigated barriers to vaccination in sub-populations with lower-than-normal vaccine coverage [2]. For example, some studies have shown that maternal health care utilization, knowledge about vaccine schedules, and the availability of health care centers and social networks are determinants of childhood immunization uptake [6–8]. Furthermore, migrant populations appear to have disproportionately low childhood vaccine uptake. For mobile populations, following through with vaccine schedules that must be taken over a period of time or in a particular order can be even more difficult than in less mobile populations. The implication is that migrant populations may have worse health outcomes either through a lack of vaccination or through inappropriate timing of vaccine schedules. More research into the vaccination coverage of migrant populations is therefore warranted.

Within Southeast Asia, Thailand has been relatively successful with regard to immunization programs, policies, and practices. The National Immunization Program (NIP) was introduced in Thailand in 1977 [9], and since 2005 the NIP has achieved immunization coverage of around 96–99 % among Thai children [10]. In neighboring Myanmar, the Expanded Program on Immunization (EPI) was launched in 1978 in 104 townships and then expanded to cover almost all areas of all 305 townships by 1997. However, the national immunization coverage varies widely (from 38 to 93 % in 2012) because of limited health infrastructure and funding, accessibility to services, population movement and difficult-to-traverse terrain [11].

Thai public hospitals, non-governmental organizations, and community-based organizations have played an important role in the provision of general health care and vaccines to cross border and migrant populations along the 2000 km Thailand-Myanmar international border [9].However, large disparities in vaccination coverage remain between migrants and native Thais. In 2013 a study exploring vaccine coverage among children of Myanmar migrants living in Bangkok revealed that the rates of complete vaccinations were much lower when compared to both Thai children and Myanmar children living in their home country [12]. For example, coverage of Bacille Calmette-Guerin (BCG) among children of Myanmar migrants age 1 was 82.6 % while the coverage for Thai children was 99.9 % and for Myanmar children was 93 % respectively. Language barriers, low levels of awareness and limits placed on mobility among migrants (because of their legal status) resulted in low immunization uptake. In 2012, a measles outbreak (without fatalities) was reported in temporary shelters in Tak, Ratchaburi and Mae Hong Son Provinces [13]. These examples highlight the necessity of targeting migrant populations in Thailand and of strengthening migrant-friendly vaccine services in all countries [13].

Recent estimates indicate that there are more than 3.5 million non-Thais living in Thailand, including documented and undocumented migrant families [14]. Among 1.3 million international migrants who held work permits in Thailand in 2009, 82 % were from Myanmar [14]. In addition, approximately 116,000 displaced persons from Myanmar are living in nine temporary shelters along the Thailand-Myanmar border [15]. In Tak Province alone, there are an estimated 27,000 registered and 200,000 unregistered migrants from Myanmar working in various industries including factories, construction, and agriculture [16].

**Fig. 1:**
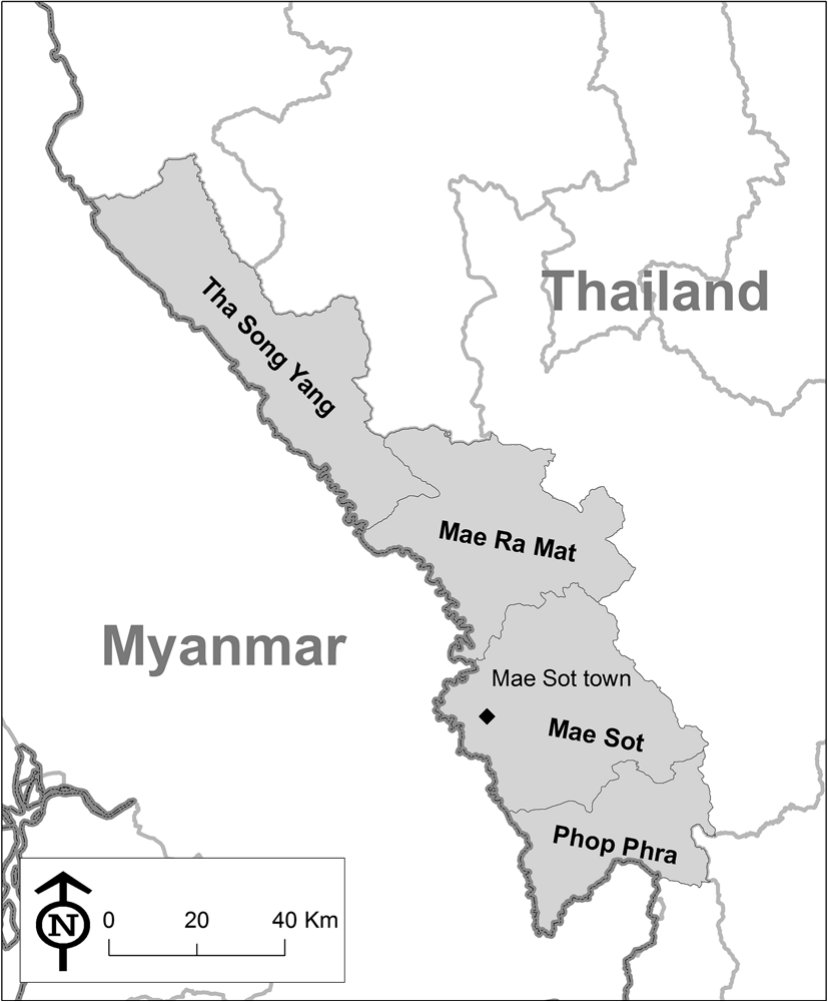
Locations of migrant schools in this project, by district of Tak Province, Thailand

These migrants aren’t solely adults. Migrants travel to Thailand with their families or establish families after arriving. Given their transitory nature and their typically impoverished and undocumented status, the children of such families are easily missed by educational and public health programs. In order to address these issues, several migrant schools exist along the Thailand-Myanmar border, most established, funded, and run by international and community-based organizations. Such schools are primarily intended for school age children (ages 5–18 years) but many also offer daycare services for younger children. In Mae Sot, Phop Phra, Mae Ra Mat and Tha Song Yang Districts of Tak Province (Fig. 1), approximately 13,561 students were attending such migrant schools in 2013 [17]. These schools also offer a ready opportunity to approach migrant children and families with regard to facilitating important health care programs, such as vaccination campaigns to children.

Therefore, in between May 14 and July 10, 2009 the Shoklo Malaria Research Unit (SMRU) conducted focus group discussions with the parents of migrant children to gain a better understanding of the barriers to childhood immunization [18]. Several difficulties that migrant parents face with regard to immunizing their children emerged from these discussions. The act of migration, long distances to immunization services, fear of being arrested on the way to health care facilities and a lack of available time because of the necessity of work were all listed as deterrents or barriers to having children vaccinated [18].

SMRU was in a unique position to address these issues as a result of funding for vaccines through the European Union (EU). This program was part of the EU funded program entitled “Providing the diagnosis, treatment and prevention measures against malaria and other infectious diseases in the uprooted population of Tak Province, Thailand” and prioritized the needs of pregnant women and children to ensure a healthy start to life. Activities in this program included the provision of antenatal care, delivery services and vaccination campaigns through schools and clinics. In collaboration with the Tak Provincial Public Health Office and Mae Sot General Hospital, SMRU launched a school-based immunization program that provided free vaccinations to children at migrant schools in June 2009. Transportation to school was provided, minimizing the barriers and dangers that travel distances to health clinics pose.

The goal of the present paper is to retrospectively assess the relative successes and failures of this migrant school childhood vaccination project. Vaccination coverages of migrant children are seldom reported and the data frequently do not exist. Such data and reports are therefore important for better understanding the health of migrant populations and for informing future campaigns aimed at vaccinating populations that are, for a variety of reasons, difficult to reach.

## Methods

### Population

The target population for the vaccination program included migrant school children in four districts of Tak Province (Fig. 1). The migrant schools are only meant for children of migrants from Myanmar. The United Nations defines a migrant as an individual who has resided in a foreign country for more than one year irrespective of the causes, voluntary or involuntary, and the means, regular or irregular, used to migrate [19]. The study follows this definition for migrants who have migrated to Thailand from Myanmar and have been staying in Thailand for a minimum of one year but also includes short-term migrants, such as seasonal farm-workers who travel for short periods to work in the agricultural sector. Often they migrate along with their family and young.

Schools for children of migrants from Myanmar have operated for decades in Thailand due to the economic, political and educational landscape within Myanmar and because many migrants are in their reproductive years. A 2012 report by the International Organization for Migration (IOM), estimates that approximately 377,000 migrant children (under 18 years of age, and 11 % of the total migrant population) are in Thailand and that despite a government policy requiring all children in Thailand to attend primary school irrespective of their immigration status, only a small fraction of migrants actually enroll [20]. Studies further suggest that about half, or 150,000, were actually born in Thailand, where they fall under the same category as their parents and are not entitled to either long-term residence or citizenship [20]. School certificates from these schools are not officially recognized by either government. However for many migrant children, the schools have made a significant contribution to numeracy and literacy and provided some hope for a better life.

This program began in June 2009 and lasted until March 2014 (the Thai school year begins in June and ends in March). Ages of children vaccinated through the program ranged from 1 to 15 years. The SMRU vaccine team asked parents of migrant school children to provide an immunization card issued by any organization if children had previously been vaccinated. Children were included regardless of whether or not they had previously received vaccinations.

### Immunization Program

In collaboration with migrant school officials, the SMRU vaccine team created a student register at the beginning of each year. The roster included information on the immunization status (none, incomplete, full) of students and each student was provided a vaccine card which listed their current immunization status, the name of vaccines, and the date of vaccination and next follow up visit. The same information was recorded in an SMRU log-book.

While the program was running, the SMRU team visited each migrant school in the aforementioned districts once a month (during the school year) in order to follow through with vaccine schedules, to include newly arrived students and to update immunization records. The schedule was provided to each of the schools and school teachers were reminded by phone the day before the vaccination team visited. By design, the aforementioned program meant that migrant school children could be fully immunized if they attended school for a complete academic year *and* were present when the vaccine team visited. During the last 3 months of the school year the vaccination program was specifically focused on children who had previously missed the scheduled vaccination days. As the vaccination program rolled over to the next year, children with incomplete vaccinations could be subsequently immunized.

### Vaccination Schedules

The NIP stipulates the vaccination schedule for children aged <7 years (Table 1) [9]. However this project enrolled children in migrant schools who were mostly school age and unvaccinated and therefore a significant proportion were well past the point of reaching “age-appropriate” immunization. The SMRU vaccine schedule therefore sought to maintain the correct order and proper vaccination intervals following the NIP schedule and the recommendations from Tak Provincial Public Health Office (Table 2).

**Table 1:** National immunization schedule in Thailand

Age	Vaccine
At birth	BCG, HepB1
2 month	OPV1, DTP + HepB1
4 month	OPV2, DTP + HepB2
6 month	OPV3, DTP + HepB3
9 month	MMR1
18 month	OPV4, DTP4, JE1, JE2^a^
2 1/2 year	JE3
4 year	OPV5, DTP5
Over 7 year	Td 3 doses, HepB 3 doses, MMR2

^a^JE2: 1 month apart from JE1. *BCG* bacille calmette-guerin, *OPV* oral polio vaccine, *HepB* hepatitis B vaccine, *DTP* diphtheria, pertussis and tetanus vaccine, *JE* Japanese encephalitis vaccine, *MMR* measles, mumps and rubella vaccine, *Td* tetanus and diphtheria containing vaccine

**Table 2:** Minimal recommended interval of vaccine doses

Vaccine	Start to 1st dose	1st dose to 2nd dose	2nd dose to 3rd dose
BCG	0 to 15 years old	NA	NA
OPV	2 months to 15 years old	1 month	1 month
HepB + DTP	2 months to 6 years old	2 months	2 months
HepB	7 years to 15 years old	1 month	1 month
MMR	1 year to 15 years old	Minimum 1 month	NA
Td	7 years to 15 years old	1 month	6 months

*NA* not applicable

The SMRU immunization program focused on provision of vaccine doses in the primary series to children who had not previously been immunized. WHO guidelines and other studies define the primary series as Oral Polio vaccine (OPV) 1–3, Diphtheria, Pertussis and Tetanus vaccine (DTP) 1–3, Hepatitis B vaccine (HepB) 1–3, Measles, Mumps and Rubella vaccine (MMR) 1 and BCG 1 [21]. It does not include booster doses. Thus, we excluded the immunization status of OPV4-5, DTP4-5 and MMR2. The outcome of Japanese Encephalitis vaccine (JE) status was also excluded as it was not supported by the project budget. In 2009 measles immunization was not provided in combination with mumps and rubella but this was changed to MMR in 2010, hence this manuscript mentions ‘measles containing vaccines’ in reference to both of these preparations.

Vaccines were stored and transported in compliance with the cold chain system recommended by the Ministry of Public Health, Thailand [22]. The team prepared vaccine supplies in coolers with ice packs and thermometers every morning before their visit to the site. Vaccines were kept within a temperature range of 2–8 °C during their visit. Support for purchasing vaccine supplies and employing staff was possible for the European Union grant (Grant Numbers: 164.106 and 256.285).

### Data and Analysis

We entered immunization data into a database at SMRU and used the data records to retrospectively calculate completion proportions (coverage) for all children in the cohort (ages 1–15). The coverage of each vaccine was calculated as the proportion of children who received a particular vaccine within the appropriate age range and interval out of the total number of children in that particular age group. Vaccination was considered to be “age-appropriate” if it followed the NIP age schedule. Likewise, vaccination was considered “timely” if it fell within 30 days of the recommended interval(s). If a second dose was taken late, the third dosing schedule was based on the second rather than the first dose. Completion proportions were calculated for each specific vaccine as well as for two categories of vaccines: those with a single dose versus those with multiple doses. Finally, we also calculated completion proportions for immunization against seven vaccine preventable diseases (VPDs). All calculations were done using SPSS software (SPSS Inc, Chicago,II, USA).

### Ethical Considerations

This retrospective cohort study is based off of data that were collected as part of a vaccination program rather than a research study. Formal consent was therefore not obtained. No personally identifiable information were used or shared during the drafting of this report, so individual participants remain anonymous. No ethical committee approval was sought for this report.

## Results

Between June 2009 and March 2014 12,277 migrant school children were documented by the program, 51.6 % being male. Only 7.7 % (947) of these migrant school children had a pre-existing vaccination card from another organization, meaning that a vaccine routine had already begun for at least a portion of this population. BCG completion proportions at the time of first contact with the children was approximately equal in younger and older children (61 % of the children <7 years old and 61.5 % of those ≥ 7 years old). BCG is detectable because of a visible scar and is therefore not dependent on the vaccination card. The large discrepancy between those children with vaccine cards and those who had already received the BCG vaccine suggests that many might have also received other vaccines.

Furthermore, over 61 % of these migrant children were already over the age of seven, meaning that most already fell outside of NIP guidelines for “age-appropriate” immunization (Table 2). Of those under age 7, 42.9 % (1950/4542) received age-appropriate vaccination 57.2 % (4427/7735) when children over age ≥ 7 are included. For those already 7 years or older, we used the WHO guidelines for children with interrupted or delayed immunization and set up a schedule, with help from the Tak Public Health Office, for immunizing the migrant school children [21]. Approximately 83 % of the children (5306/6391) in this program received all of the offered vaccinations in timely manner. Around half of the migrant school children, 51.9 % (6377/12277) were fully vaccinated against the seven VPDs.

Coverage for single-dose vaccines exceeded 90 % (92.3 % for BCG and 90.9 % for measles containing vaccine). For multi-dose vaccines, coverage declined by dose. For example, the first dose of OPV vaccine was given to 98.6 % of migrant school children whereas the second and third doses were given to 91.2 and 85.3 % respectively. DTP vaccine (given to children <7 years) had the poorest coverage with 89.2, 76.2 and 63.8 % coverage in the first, second, and third doses, respectively.

## Discussion

Vaccination is one of the most important components of preventative health care. Proper timing and ordering of vaccine schedules are important for individual and population health [23–25]. However, social, economic, and political barriers to vaccination exist for some populations, leading to low levels of vaccine uptake and subsequent poor health outcomes that could be mediated if such barriers were overcome [7, 8, 26, 27]. The data presented here illustrate some of these issues.

The Thailand-Myanmar border is a region with many highly mobile and migratory people. It is a regional economic hub and a mixing point for many different ethnic groups, some of which have traditionally lived in the area since long before there were formal international borders. Receiving age-appropriate vaccines can be extremely difficult for these highly mobile, typically economically poor, people.

Furthermore, the close-quarters and sometimes population-dense settings (including schools and refugee camps) in which some migrants live or visit may create an opportune environment for the spread of diseases such as tuberculosis or meningococcal diseases. For adolescents and young adults who have not been vaccinated, the risk is probably much higher than for their vaccinated peers. The BCG vaccine can reduce the risk of developing tuberculosis (TB) by 50 % when provided at birth [28] and a single measles vaccination effectiveness for children age 12 months is estimated at 92 % (range 86–96 %) [29]. BCG and measles containing vaccine coverage in the migrant school children reached 92 and 91 %, respectively—both slightly under the Thai national coverage for both vaccines, but arguably much better than would be expected in the absence of such targeted programs [9, 30]. In 2012, measles cases were reported among displaced populations along the border, bringing increased resources for vaccination [13], however, the MMR vaccine was in short supply and therefore not available for the program between June and December 2013.

Another major difficulty with regard to vaccination and migrant populations has to do with completing successive rounds of a vaccine regime. For people who are frequently on the move, following through with a complete regimen (multiple doses spaced across a long period of time) of even a single vaccine can also be difficult, as people are likely to move before the end of the regimen. In our data the coverage of HepB, OPV, DPT, and Td decreased with increasing number of doses (Table 3), particularly for Td vaccine which requires the longest interval between the second and third doses. This pattern has also been observed in other migrant populations [7, 8, 31]. In addition, children under 7 years old were less likely to complete their required vaccines indicating school is less of an effective target point for their vaccinations.

**Table 3:** Vaccine coverage and timeliness of dose intervals for each primary dose of seven VPDs

Vaccine	Coverage % (N)	Too early % (N)	Timely %(N)	Delayed % (N)
BCG	92.3 (11,328)			
OPV1	98.6 (12,108)			
OPV2	91.2 (11,202)	0.2 (25)	92.8 (9869)	6.9 (738)
OPV3	85.3 (10,475)	0.1 (9)	92.1 (9216)	7.8 (777)
DTP1	89.2 (4051)			
DTP2	76.2 (3462)	11.0 (369)	66.6 (2236)	22.4 (752)
DTP3	63.8 (2898)	13.5 (372)	86.1 (2365)	0.4 (10)
Td1	97.8 (7561)			
Td2	91.9 (7107)	0.3 (18)	94.2 (6282)	5.5 (368)
Td3	60.7 (4692)	3.3 (143)	93.8 (4127)	3.0 (130)
HepB1	96.5 (11,843)			
HepB2	87.2 (10,701)	0.2 (15)	91.0 (8875)	8.9 (867)
HepB3	72.2 (8859)	0.0 (3)	99.8 (8061)	0.2 (17)
Measles containing vaccine	90.9 (11,159)			
Full Immunization	51.9 (6377)			

Timely: recommendation varies with vaccine (Table 2)

By 2015, the ASEAN Economic Community (AEC) plans to free movement of goods, services, investment, and capital within the region [32]. This could lead to increased movement of people across the international border and potentially to increased challenges for infectious disease control. Thus, immunization in vulnerable populations such as migrants will be an important goal for the control of vaccine-preventable disease.

Documenting vaccination successes and failures in migrant populations is also difficult, since different organizations and institutions have different approaches to record keeping. The Ministry of Public Health in Thailand, NGOs and CBOs provide multi-language vaccine cards to migrant children [9, 17], however few migrant school students in our program were able to present a vaccination card. For some, this is an indication that they had not been provided vaccinations. In other cases this may be the result of losing the cards or documents being left in Myanmar and not available in Thailand.

Given these problems, a registration system for migrants in Thailand could help strengthen health information systems and surveillance. For example, a strategy which incorporates a unique identity number for the migrants and their family members could be used for digitizing information regarding vaccination and other health-related histories. However, undocumented migrants may avoid such a system since their legal status is questionable and since they are at risk of deportation punishment by law enforcement officials. Currently, public hospitals in Tak Province provide vaccination to migrant children and pregnant women through outreach services regardless of legal migrant status. Limited language proficiency and legal status among migrants, as well as a lack of awareness of available services, all pose challenges to accessing these immunization services. Migrant friendly vaccination services which address these issues need to be strengthened in order to achieve better health outcomes for migrant children.

It is also essential to raise awareness among health professionals of migrants’ rights to health and vaccination in Thailand and Myanmar. International support from agencies such as the EU has had enormous benefits for population health, in this case by contributing to improved immunization coverage in a marginalized population. This highlights the potential advantages of a vaccination program that is implemented in a systematic fashion, through routine immunization and data recording, working through both national health systems and in collaboration with international support and NGOs. Although the Thai government has initiated progressive reforms to improve the welfare of migrant children, (for example in 2010 the government allowed the children of migrants to register and buy health insurance on a voluntary basis), few migrant workers have utilized this option [20]. The International Organization for Migration has called for significant reform and dialogue in this arena for the development of Thailand and Myanmar [20].

Another potential solution for this problem could be a biological test for previous vaccination [33, 34]. Such a test would need to be able to differentiate between previous infection(s) and vaccine(s), and would need to be cheap relative to the cost of vaccination [35]. If such a test were not prohibitively expensive, it could provide a cost-effective approach toward knowing a patients’ vaccine history regardless of the absence or presence of vaccine documents, and could therefore help the caregiver make informed and economically sound public health decisions as to whether or not to provide a vaccination.

There are several limitations to our report. We were unable to adequately estimate prior dosing and vaccination, therefore migrant school children were categorized as unvaccinated if they were unable to present the vaccination card. Furthermore, migrant school children are unlikely to be representative of the true population (migrant children) at risk and we only able to report on those in schools [14, 36]. Finally, while there were no reports about seizures in measles vaccine (MMR or measles) recipients, there was no formal reporting system. This is important given a recent report of the increased incidence of post-vaccination seizures if vaccination of measles vaccine (MMR or measles) is delayed past 15 months of age [37].

Regardless, given the dearth of information about vaccination in migrant children it is important to document and report these experiences so that vaccine programs can be informed and improved. Very few of the children we reached in the migrant schools appeared to have previously received adequate vaccination. The children that we were unable to reach are probably even less likely to have received adequate vaccination, meaning that we have only scratched the surface of this problem. However, this program did show the utility of reaching out to children attending migrant schools, and that given sufficient funding and supplies such children can receive timely vaccination in this setting.

Aside from expanding coverage in these populations, future efforts should seek to improve vaccination documentation including any potential side effects, or alternatively find a biological test for vaccine history. Finally, cross-border dialogue and continued integration of health care providers should be strengthened to improve immunization efforts in migrant children. Collaborations across international borders are crucial for improving the health of populations who exist across those borders.
